# Cibacron Blue F3GA ligand dye-based magnetic silica particles for the albumin purification

**DOI:** 10.55730/1300-0527.3599

**Published:** 2023-10-10

**Authors:** Nurhak TATAR, Semra AKGÖNÜLLÜ, Handan YAVUZ, Adil DENİZLİ

**Affiliations:** 1 Institute of Nuclear Sciences, Hacettepe University, Ankara, Turkiye; 2 Division of Biochemistry, Department of Chemistry, Hacettepe University, Ankara, Turkiye

**Keywords:** Albumin purification, Cibacron blue F3GA, ligand dye, magnetic silica particles, protein purification

## Abstract

Dye-ligand affinity chromatography is among the increasingly popular affinity chromatography based on molecular recognition for the purification of albumin. This study focuses on the binding of Cibacron Blue F3GA ligand dye with magnetic silica particles and purification by separation. Mono-disperse silica particles with bimodal pore size distribution were employed as a high-performance adsorbent for human serum albumin (HSA) protein purification under equilibrium conditions. The synthesized ligand-dye affinity based magnetic silica particles were characterized by electron spin resonance, Fourier-transform infrared spectroscopy, scanning electron microscopy, vibrating sample magnetometer, elemental analysis, and dispersive X-ray analysis. The HSA purification performance of the proposed material in the presence of a magnetic field was relatively investigated using magnetic-based particles with similar morphologies. The maximum adsorption capacity for HSA in an artificial plasma medium was defined as 48.6 mg/g magnetic silica particle. By using the designed magnetic silica particles, 1.0 M NaCl solution was successfully utilized for obtaining quantitative desorption with HSA. However, continued HSA purification performances of magnetic-based particles were significantly lower concerning the ligand-dye magnetic silica particles. The purity of the removed albumin was about 97%. The magnetic silica particles could be utilized many times without decreasing their protein adsorption capacities remarkably.

## 1. Introduction

The strategies for effective and large-scale purification of proteins are significant for protein biosynthesis and analysis. Affinity-based techniques, which enable highly selective and efficient purification of a single protein through specific molecular recognition and binding to its ligand, have been commonly applied for a variety of protein purification [1]. There are many different types of proteins in the blood serum or plasma proteome, which is a gold mine for disease biomarkers [2]. With an increasing market demand, human serum albumin is an essential therapeutic agent and disease biomarker [3]. Albumin consists of a single chain of 585 amino acids and has a molecular mass of 66.5 kDa [4–8]. Approximately 60% of human plasma protein contains human serum albumin (HSA). HSA has many important roles such as transporting molecules, conservation of osmotic pressure, stabilizing extracellular fluid volume, capillary membrane permeability, and the effect of neuroprotective items in human plasma. It is also used broadly such as drug delivery, carrying, and transporting molecules in pharmaceutical sciences [9–17]. Many techniques are available for the separation and purification of albumin [18–22]. For example, classical Cohn’s method which is widely used, plasma fractionations, precipitation from ethyl alcohol, purification from the placenta, simulated moving bed chromatography, ion exchange chromatography, column chromatography, liquid chromatography, heat shock method, and affinity chromatography (immobilized metal-ion, boronated, immunoaffinity and dye-ligand affinity). Among these methods, the dye ligand affinity method provides highly selective, high adsorption capacity, efficient purification of an individual protein, easy immobilization, and large-scale separation. Because of their group-specific ligands, dyes interact with many proteins by mimicking their structures. There are some difficulties with classical dye affinity chromatography including slow flow rate in the column and contaminating of the column. To solve these difficulties magnetic separation methods obtain different options [23].

Magnetic nanoparticles are one of the most established nanomaterial species because of their biocompatibility in the quickly growing fields of nanobiotechnology. Silica is the best choice for coating because of its features including biocompatibility, nontoxicity, chemical inertness, and biostability. Additionally, magnetic mesoporous silica particles demonstrate favorable physical and chemical characteristics as an adsorbent, especially due to their related advantages in large surface areas and diffusion rates, low toxicity, chemically modifiable surface, and ease of separation under external magnetic fields. They also exhibit attractive characteristics, such as the possibility of controlling their particle size, the chance of functionalization of the silica surface, biocompatibility, degradability under physiological conditions, and large surface areas and pore volumes [24,25]. One more advantage of silica coating is that the terminated silanol groups on the surface can be modified with varied coupling agents to covalently attach specific ligands to the surfaces of these magnetic polymers [26]. As a result magnetic silica particles have been of great interest for years due to their practical importance in various fields. In recent years, the emerging field of in vitro and in vivo diagnostics, analysis, and measurements within intact biological systems such as tissues, blood, and single cells with the employment of nanoparticle tools has attracted great interest [27]. It can be considered that both surface chemistry and porous features of silica adsorbent are the most influential factors controlling the reversible adsorption/desorption behavior and subsequent isolation efficiency of protein. The porous characteristics of silica polymer particles such as average porosity, pore size, pore size distribution and specific surface area can be tuned by controlling synthesis conditions. The surface chemistry of adsorbent depends predominantly on both the kind of base material and ligand employed for derivation of the base material [28, 29]. It focused on the synthesis of silica adsorbents in form of monodisperse-porous particles with various chemistries and porous characteristics. In this study, magnetic silica particles with ligand Cibacron Blue F3GA were synthesized, and albumin purification was carried out in the presence of a magnetic field. For this purpose, firstly magnetic silica particles were synthesized, and Cibacron Blue F3GA (CB – F3GA) dye ligand was covalently attached to these magnetic particles. Dye-affinity magnetic silica particles (CB–F3GA–m–Silica) were characterized by Electron spin resonance, Fourier-transform infrared spectroscopy, scanning electron microscopy, vibrating sample magnetometer, elemental analysis, and dispersive X-ray analysis. Then albumin adsorption studies from aqueous protein solutions and human serum were carried out. Elution of albumin and reusability of these dye-affinity adsorbents were also investigated.

## 2. Materials and methods

### 2.1 Chemicals

Tetraethyl orthosilicate (TEOS), tetrabutylammonium iodide (TBAI), 2–propanol (IPA), tetrabutylammonium iodide (TBAI), ammonium hydroxide was purchased from Sigma Aldrich Chem. Co., U.S.A. Human serum albumin (HSA, 98% pure by gel electrophoresis, 67 kDa) was obtained from Aldrich Chem. Co. (Milwaukee, WI, USA). Cibacron Blue F3GA was obtained from Polyscience (Warrington, USA).

### 2.2 Synthesis of magnetic silica particles

A brief definition of silica–coated magnetic particles polymerization method is as follows [30]. The fixedly positioned iron oxide particles were produced by precipitation of iron salts into the porous inner structure of poly(methacrylic acid–co–ethylene dimethacrylate) (poly(MAA–co–EDMA)) in an alkaline medium. The synthesis of silica-coated iron oxide particles was based on the stepwise hydrolysis and condensation of polymethacrylate particles containing iron oxide particles. After mixing 30 mL of 2–propanol, 7.5 mL of distilled water, 0.4 mL of 0.1 M NH_4_OH solution, 375 mg of TBAI was dissolved in the solution. Polymethacrylate particles containing 0.5 g of iron oxide were dispersed by ultrasonication at 180 W, 50 Hz for 1 min and stirred for 30 min. 3.0 mL of 2–propanol and 1.5 mL of TEOS solution were dropped into the prepared iron oxide solution and stirred overnight in a stirrer at room temperature. With the help of a magnet, the silica– gel polymethacrylate particles formed in the solution were separated. The separated particles were washed with ethanol and distilled water and dried under vacuum at 70 °C for 48 h. Silica-coated iron oxide particles with porous structure were obtained as a result of the particles pulverized under atmospheric pressure for 2 h at a heating rate of 450 °C at 2 °C per min.

### 2.3 Preparation of CB - F3GA attached magnetic silica particles

The monochloride-triazine dye Cibacron Blue F3GA, used as an affinity ligand, was covalently bound to the particles. In this procedure, 100 mg of silica-coated magnetite (Fe_3_O_4_) was first used. Different amounts of Cibacron Blue F3GA (0–5 mg/mL) were dissolved in a 10 mL solution containing 0.1 M NaOH to prepare a total of 100 mL dye solution. The prepared solution was added to the particles and the binding reaction was carried out at 80 °C for 4 h. After the reaction, unbound Cibacron Blue was washed with distilled water to remove F3GA residues and then precipitation was performed. The magnetic particles were dried using a lyophilize (Christ alpha 1–2 LD, Germany) with freeze-drying for characterization.

### 2.4 Characterization of magnetic silica particles

#### 2.4.1 Scanning electron microscopy

The surface morphology of the magnetic silica particles was investigated by scanning electron microscopy (SEM; Zeizz, EVO 50, German). The sample was first subjected to the coating process and then SEM images were obtained at different magnifications.

#### 2.4.2 Surface area analysis

Surface area measurements (Quantochrome, Nova 2200e, USA) of magnetic silica particles were performed by adsorption and desorption process at 210 °C and 100 °C in nitrogen atmosphere and the data obtained were calculated.

#### 2.4.3 Electron spin resonance

The ESR spectrum of the magnetic silica particles was determined with an ESR analyzer (Bruker EMX 113X-Band). The results obtained using the absorption curves of the ESR spectra under the conditions were given.

#### 2.4.4 Vibrating sample magnetometer (VSM)

The magnetic properties of magnetic silica particles were also determined by another method, vibrating sample magnetometry (Quantum Design Model 600). Measurement conditions were carried out at room temperature, under an intensity of ±20 kOe, and in steps of 50 Θe. Also, the measurement sensitivity was 1 μemu.

#### 2.4.5 Elemental analysis

The quality of sulfur determined using the elemental analysis belongs to Cibacron Blue F3GA triazolyl dye that was attached to the magnetic silica particles (1.0 mg) were placed in the aluminum sample cell of the elemental analyzer (Leco, CHNS - 932, USA), hence magnetic silica particles did not involve any sulfur in their chemical structure. Also weighed with an accuracy of ±0.0001 g.

#### 2.4.6 Dispersive X-ray analysis

The presence of Si and Fe atoms, which are the basic components of the structure, was investigated by EDX analysis of magnetic silica particles attached to Cibacron Blue F3GA.

#### 2.4.7 Fourier transform infrared spectroscopy

Two mg samples were taken from the dye-free and Cibacron Blue F3GA attached magnetic silica particles, placed in an attenuated total reflected Fourier transform infrared spectrometer (ATR - FTIR, Thermo Fisher Scientific, Nicolet iS10, Waltham, MA, USA) and the spectrum was recorded in the range of 400–4000 cm^−1^.

### 2.5 Protein analysis

**Figure 1:**
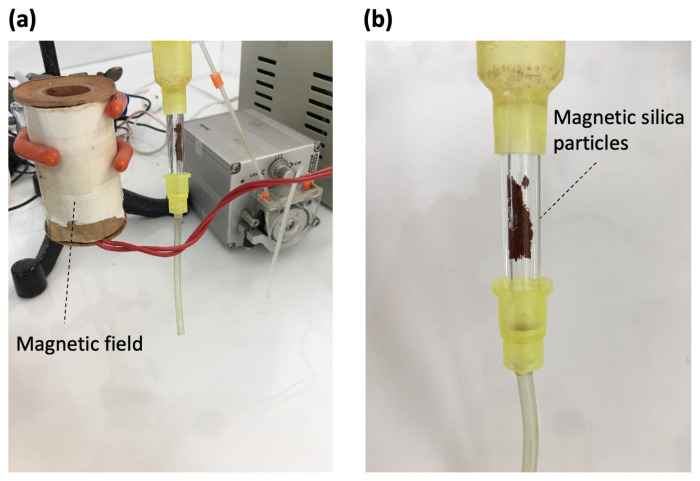
(a) Magnetic field; (b) magnetic silica particles.

Adsorption of HSA from human plasma with Cibacron Blue F3GA-attached magnetic silica particles was carried out on a magnetic field platform. The parameters affecting the adsorption capacity were investigated with temperature, time, pH, initial concentration, dye-ligand amount, effect of magnetic field. To see the effect of pH and temperature parameters on adsorption capacity firstly buffer systems in the range of 4.0–8.0 for pH effect and secondly for temperature effect in the range of 4 °C–45 °C ambient temperature was used in this investigation. Besides, for the effect of initial concentration of HSA ranging from 0.01 to 3 mg/mL was used. The optimized binding buffer was chosen 0.1 M acetate phosphate at pH 5.5 at 25 °C and the optimal elution agent was used 1.0 M sodium chloride. Finally, HSA binding capacity was investigated using both silica and dye-attached silica particles. The total protein content in both human serum and plasma samples was determined at 280 nm using a UV-Vis spectrophotometer (Shimadzu, Model 1601, Japan). The experimental setup used to study adsorption of albumin in the presence of a magnetic field is shown in Figure 1.

#### 2.5.1 Regeneration of magnetic silica particles

1.0 M NaCl solution was used as desorption agent to break secondary interactions to desorb protein residues adsorbed from magnetic particles bound to CB–F3GA. In this process, the desorption solution was added to the sample and shaken for 2 h at room temperature.

#### 2.5.2. Protein quantitation and analysis of purity

Sodium dodecyl sulfate–polyacrylamide gel electrophoresis (SDS–PAGE) was performed to evaluate the protein samples purity. Samples were applied in a mini-protein TGX Stain-Free Precast Gels and ran at 150 mV.

## 3. Result and discussion

### 3.1 Characterization results

**Figure 2:**
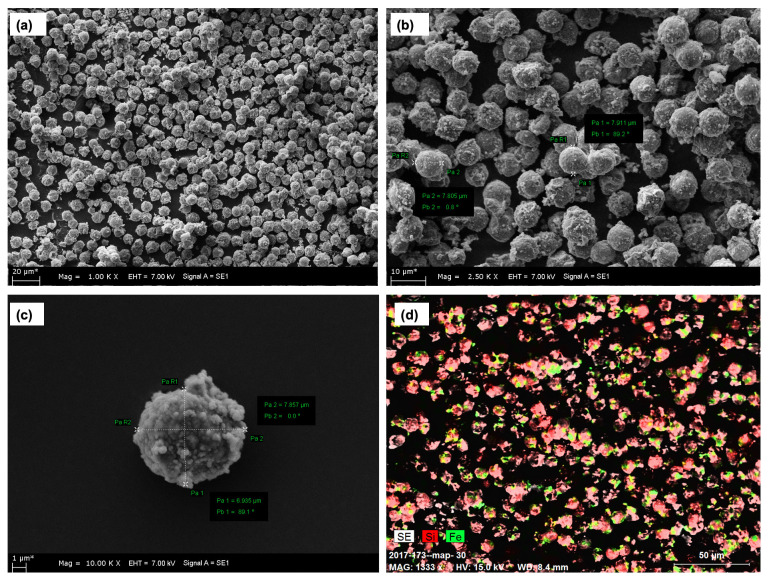
SEM images of different magnification of structures: (a) 1.00 KX; (b) 2.50 KX; (c) 10.00 KX; (d) 1333 X maping (Fe, colored green; Si, colored red).

The surface morphology of the dye affinity magnetic silica particles was studied using SEM and is shown in Figure 2. The particles were clearly shown to have spherical shapes with rough surfaces. It is also seen that they have a narrow size distribution, and the sample size averages around 7.8 μm. Another inspection of the SEM instrument, mapping, is shown in Figure 2. The silica and iron structures contained in the Cibacron Blue F3GA doped magnetic silica particles can be seen. The porosity distribution was obtained by nitrogen adsorption/desorption method. The results are given in Table 1. According to these results, the average size of the pore structure was 41 nm and macropores and mesopores appeared in the structure. The specific surface area of the magnetic silica particles was found to be 222 m^2^/g. This value indicates that the particles have a high surface area, thus indicating the binding of a sufficiently high number of iron oxides to the silica mesopores. It can be concluded that the size distribution coefficient of variation (CV) of magnetic silica particles has a narrow range of 4.5%. This value indicates the existence of a structure in which the range of size distribution variation is not high.

**Figure 3:**
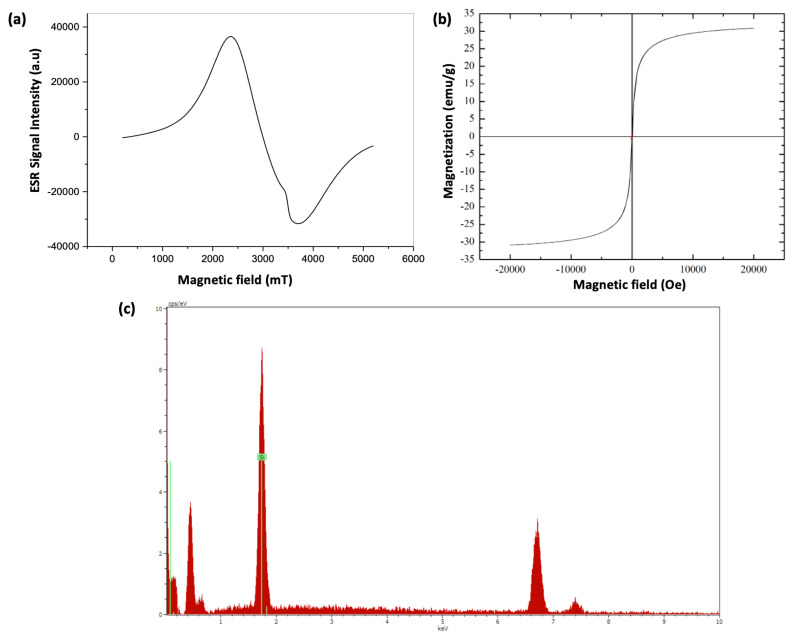
ESR spectra of magnetic CB – F3GA – m – Silica (a); VSM spectra of magnetic CB – F3GA – m – Silica (b); EDX spectra of magnetic CB-F3GA – m – Silica (b).

The presence of magnetic particles in the structure was confirmed by electron spin resonance (ESR) spectroscopy. The spectrum of the magnetic peaks against the magnetic field is shown in Figure 3(a). The g value, which is the important spectroscopic parameter obtained when resonance conditions are met, was found to be 2.3153. Another spectroscopic parameter derived from the spectrum is the peak-to-peak line width DH_pp_ @ 135 mT. Previous studies have shown that the range of variation of the g constant for Fe(III) ions in low spin intensity structures is between 1.4–3.1 and the range of variation of the g constant for Fe(III) ions in high spin intensity structures is between 2.0–9.7 [31]. Since the determined g value of the examined sample takes into account the reference values, it can be said that Fe complexes showing ferrimagnetic properties are present in the particle. When the spectrum is examined, in addition to the broad signal with ferrimagnetic Fe atoms, another signal formation around 3500 G (g ~ 2.002) with lower intensity and paramagnetic character is observed. This signal may belong to the polymer on which the iron atoms are coated, or it may be due to the materials and/or impurities used in the preparation of the sample.

Magnetic field dependence of magnetization, M(H) was measured at 300 K for sample magnetic CB–F3GA particles as shown in Figure 3(b). According to the results, the saturation magnetization of the particles was observed as 30 emu/g. Such a high magnetic saturation value implies strong magnetic responsivity of the magnetic sample. Also, no coercivity was observed at any value. It could be said that there was no forcing field in the particles at room temperature and when we consider the high magnetization value, the magnetic sample exhibits a superparamagnetic property in which the spins are randomly oriented, and no remanence remains once the applied magnetic field was removed.

The amount of Cibacron Blue F3GA bound to magnetic silica particles was investigated using sulfur stoichiometry. The amount of dye ligand attached to magnetic silica particles interacting with different concentrations of dye solutions is given in Table 2. After binding of Cibacron Blue F3GA to magnetic silica particles, an increase in all N, C, H, and S atoms in the structure is observed. This indicates that these atoms originate from the bound dye, hence the presence of the dye. It was also observed that the amount of dye bound to the particles increased with increasing CB–F3GA dye concentration. As seen in Figure 3(c), the silica atoms in the structure clearly show themselves while providing information about the content of the particles in the spectrum resulting from EDX analysis. Thus, the silica coating status in the structure was also determined.

The FTIR analysis spectra of the dye-attached magnetic silica particles and magnetic silica particles are given together in Figure S1. 3293 cm^−1^ and 1053 cm^−1^ represent symmetric and asymmetric stretching vibrations of the silanol groups in the structure. This is an indication that iron oxide particles are bound to silica. The electrostatic interaction between iron ions and −OH groups also contributed to the interactions of H bonds within the band hydroxyl groups seen at 1645 cm^−1^ wave number. All other peaks between 790 cm^−1^ and 410 cm^−1^ show vibrational bands between iron and oxygen. This is evidence of the magnetic property of the structure. The spectrum of all bands is shown in Table 3. The peak wavelength of 1594 cm^−1^ represented the stretching vibration between carbon and nitrogen atoms in the triazine ring of the dye. The peak at 1355 cm^−1^ indicates the stretching vibrations between S and O atoms and was a clue for the binding of Cibacron Blue F3GA to the structure. The biggest evidence of Cibacron Blue F3GA binding is the C – Cl stretch vibration band in the Cl – containing amino benzene ring, which we can see at 1057 cm^−1^. This band, along with other weakly intense bands in the 592 cm^−1^–406 cm^−1^ band range, can be taken as an indication of the incorporation of Cibacron Blue F3GA dye into the structure.

### 3.2 Protein analysis

The amount of ligand loading is an important parameter in the affinity separation of proteins. To determine the effect of the amount of Cibacron Blue F3GA bound in this study, the effect of the amount of dye bound to magnetic silica particles interacted with different concentrations of dye solution on HSA adsorption is given in Figure S2. As can be seen in the figure, the amount of HSA bound increases with the increase in dye concentration up to 3 mg/mL, while at higher dye concentrations, there is no significant increase in the amount of dye bound and thus the amount of HSA bound. This can be explained by the saturation of the groups on the surface to which the dye ligand can bind. Therefore, magnetic silica particles dyed with 3 mg/mL Cibacron Blue F3GA solution were used in other studies.

**Figure 4:**
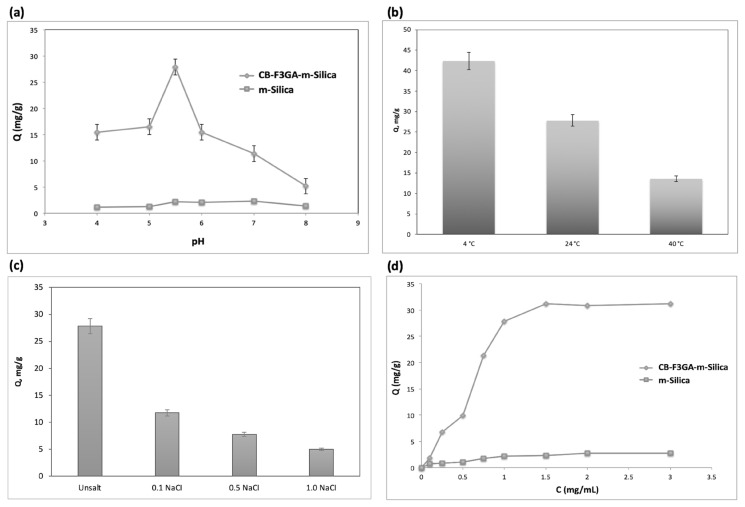
(a) Effect of pH for HSA adsorption: C: 1.0 mg/mL; time: 120 min; (b) Effect of temperature for HSA adsorption: (c) Effect of salt type for HSA adsorption: C: 1.0 mg/mL; pH: 5.5; time: 120 min; (d) Adsorption of HSA on magnetic silica particles of pH: 5.5; V_total_: 5.0 mL; 0.1 mg polymer; time: 120 min.

HSA adsorption on magnetic silica particles attached to Cibacron Blue F3GA was investigated between pH 4.0–8.0. As shown in Figure 4(a), HSA adsorption increased with increasing pH and the highest adsorption amount was recorded at pH 5.5 (27.82 mg/g). HSA adsorption was observed to decrease at higher pH values. The electrostatic force, one of the adsorption interactions, showed itself more clearly at varying acidic and alkaline conditions. Therefore, since the HSA molecule was more positively charged in the interaction up to the highest point, the electrostatic attraction between the charged groups of Cibacron Blue F3GA increased. Thus, the pH value to be used for adsorption studies under other conditions was determined as 5.5 (acetate buffer).

To investigate the effects of temperature on HSA adsorption on magnetic silica particles attached to Cibacron Blue F3GA, studies were carried out at 4 °C, 25 °C, and 40 °C temperature values. The results obtained are given in Figure 4(b). It was observed in Figure 4(b) that the increase in temperature decreases the amount of HSA adsorption. The hydrophobic contributions resulting from the interactions between the protein and the ligand become more pronounced with increasing temperature and change proportionally with the increase. The increase in temperature causes the protein to rearrange by acting on its native structures and therefore affects the adsorption capacity. Moreover, since the adsorption process is an exothermic interaction, it tends to decrease inversely proportional to the increase in temperature. In this study, it was observed that temperature increase decreases the amount of adsorption. Therefore, it can be said that electrostatic interactions rather than hydrophobic interactions are dominant in the interaction between Cibacron Blue F3GA and HSA molecules.

The effect of the ionic strength of the medium on HSA adsorption was studied using adsorption media containing unsalt, 0.1 M, 0.5 M, and 1.0 M NaCl. The results obtained are given in Figure 4(c). As a result of the studies, it was observed that the increase in ionic strength had a decreasing effect on the adsorption capacity. The highest adsorption amount was obtained in the salt-free solution. By increasing the salt concentration from 0.1 M to 1.0 M, the adsorption amount decreased by 58.3%. The negative effect of salt on adsorption can be explained by the increase in ionic strength which increases the presence of Na ions in the solution, which reduces the interactions between the ligand and the protein by accumulating on and neutralizing the sulfonate groups on Cibacron Blue F3GA. Another effect contributing to the decrease in the amount of adsorption is the change in the native structure of the protein with the increase in ionic strength. Another way in which the increase in ionic strength was reflected in the interaction between protein and ligand was the deformation of existing salt bridges in the ionic strength.

To study the effect of HSA concentration on adsorption, HSA solutions with concentrations ranging from 0.1–3.5 mg/mL interacted with magnetic silica particles bound to Cibacron Blue F3GA at pH 5.5 and the results are given in Figure 4(d). As seen in Figure 4(d), the amount of adsorbed HSA increased with the increase in HSA concentration. At an initial concentration of 1.5 mg/mL HSA, the amount of adsorbed HSA reached its highest value (34.9 mg/g) and after this value, the increase in concentration had no significant effect on the adsorption amount.

The comparison of the adsorption value examined in the presence of magnetic field is given in Figure S3. While adsorption was 6.8 mg/g with silica coated magnetic particles, this value reached 27.8 mg/g because of Cibacron Blue F3GA binding. In the presence of magnetic field, it was observed that the same particles provided HSA adsorption more effectively and reached 34.9 mg/g. This result can be explained in two ways; (i) the dissociation rate of protein and ligand in the continuous system is higher than the binding rate in the magnetic system; (ii) the ligand-dye Cibacron Blue F3GA is present both on the surface and in the pores of magnetic silica particles. The presence of flow and magnetic field facilitates the access of proteins to the pores and thus increases the amount of adsorption.

For the determination of albumin adsorption from artificial plasma, albumin determination in plasma samples after adsorption was carried out colorimetrical with Olympus AU 2700 (Mishima, Japan) analyzer. Accordingly, the amount of serum albumin bound to magnetic particles attached to Cibacron Blue F3GA was determined as 48.6 mg/g. Considering the total protein amount, approximately 97% of protein adsorption from artificial plasma was realized as albumin.

### 3.3 Reusability

**Figure 5:**
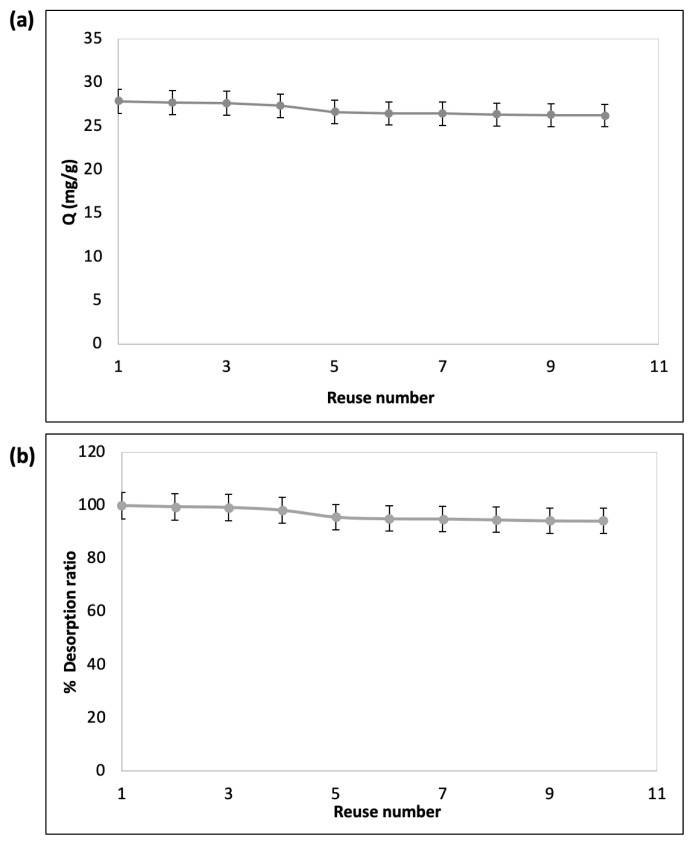
(a) The reusability of m–silica particles in terms of adsorption amount; and (b) desorption ratio%: C: 1.0 mg/mL; pH 5.5; time: 120 min; desorption agent: 1.0 M NaCl, temperature: 25 °C.

The reusability of CB–F3GA–m–silica particles was studied by applying HSA samples of 1.0 mg/mL for 10 cycles (Figure 5(a)). According to these results, it was determined that the adsorption capacity of HSA was approximately 95% after each desorption process and therefore the reduction was around 5%. This means that 1.0 M NaCl solution is a good desorption agent for the adsorbent and the prepared particles also showed good stability and significant success in terms of efficiency in reuse (Figure 5(b)).

Table 4 shows the comparison of available chromatography techniques modified with different adsorbent types. Various parameters such as polymer, method, adsorption capacity and actual sample are presented to study the purification methods. Liu et al. developed polymeric ionic liquids (PILs) PIL–G/SiO_2_ nanocomposites to investigate the adsorption behaviors of bovine serum albumin (BSA) [32]. Bhacta et al. prepared synthetic nanopockets on magnetic nanoparticle surfaces and reported that the HSA binding capacity of the material was 21 mg/g [33]. Dye-ligand attached magnetic poly(vinyl alcohol) (mPVAL) for human serum albumin (HSA) adsorption from human plasma was developed by Odabasi. The HSA adsorption value of The Cibacron Blue F3GA attached mPVAL beads was 88.7 mg/g [34]. Recently, Wang and coworkers prepared a novel highly specific bovine serum albumin cobalt–molybdenum double-hydroxides surface-imprinted composite particles (Co–Mo LDHs@MIPs). The adsorption capacity of Co–Mo LDHs@MIPs was calculated as 423.37 mg/g for BSA [35]. In this study, Cibacron Blue F3GA-attached magnetic silica particles prepared a good stable and biocompatible adsorbent matrix for simple, rapid, inexpensive, and active purification of HSA.

### 3.4 HSA purification from human plasma

HSA adsorption by magnetic silica particles attached to Cibacron Blue F3GA was further investigated in artificial plasma. Artificial human plasma was supplied by Tokra Medical (Ankara, Turkey). MTI™ control plasmas are obtained from normal plasma of human origin. HSA was added to the artificial plasma at a total albumin concentration of 1.0 mg/mL. Sodium dodecyl sulfate-polyacrylamide gel electrophoresis (SDS-PAGE) was used to determine the purity of purified HSA. The mini-protein TGX Stain-Free Precast Gels were used in this process. After this process, the gel was stained with Coomassie Blue G-250 stain for 12 h and then washed with a solution containing 20% methanol, 20% acetic acid, and 60% water.

**Figure 6:**
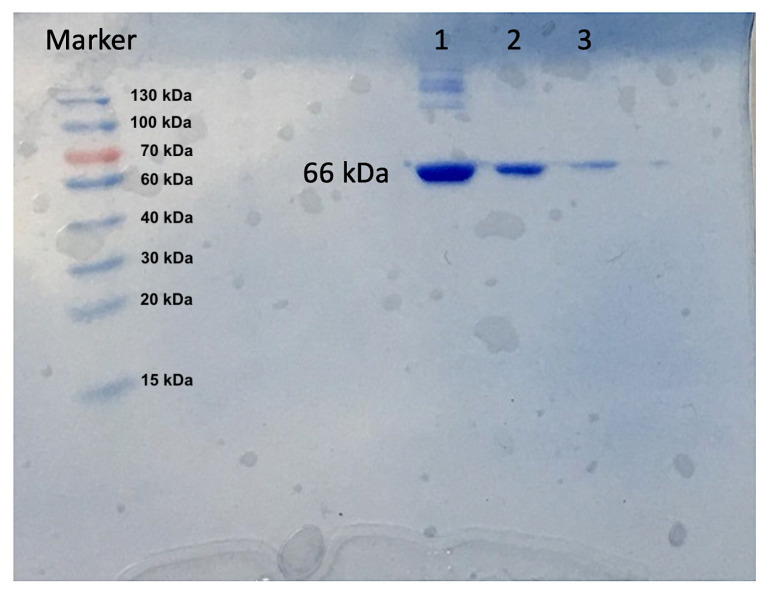
SDS–PAGE for Albumin: Lane 1: artificial plasma; Lane 2: desorption; Lane 3: adsorption.

In Figure 6, the number 1 column shows the 1/3 diluted artificial plasma, the number 2 column shows the artificial plasma after desorption, and the number 3 column represents the artificial plasma after adsorption. In the column representation of the postadsorption plasma, the albumin concentration on the molecular weight scale has decreased and has become almost indistinct.

### 3.5 Kinetic models

Kinetic models have been an important approach in determining quantities such as chemical reactions and mass transfer in adsorption processes in two ways, first and second order. For this purpose, pseudo-first order and pseudo-second order models have been used. Lagergren’s first order rate equation was the most practical approach used to study adsorption for solutes in any solution. The mathematical model of this approach is given in Equation (1). The kinetic models are given in Figure S4.

A comparison of the theoretical value of Q_e_ for pseudo-first-order and pseudo-second-order rate laws was determined to explain the adsorption kinetics and it was found that the pseudo-second-order kinetic model was in good agreement (Table 5).

### 3.6 Adsorption model

Adsorption isotherm models were studied to explain the interactions of proteins with adsorbents. For this purpose, Freundlich and Langmuir isotherm models were calculated (Table 6). Langmuir adsorption isotherm is a model in which molecules interacting with binding sites of similar energies bind only in a monolayer with equal energy. Therefore, it was observed to have a homogeneous distribution. Possible interactions of molecules with other sites are not included. The Langmuir and Freundlich approach are given in Equations (3) and (4):

Langmuir equation


(3) $$\frac{C_{eq}}{Q}=\left\{\frac{1}{Q_{max}\times b}+\frac{C_{eq}}{Q_{max}}\right\}$$

Freundlich equation


(4) $$\text{ln}Q_{eq}={LnK}_{F}+\frac{1}{n}C_{eq}$$

When compared in terms of correlation coefficients, this study fits more to the Langmuir adsorption approach as shown in Figure S5 and the maximum adsorption value is closer to the experimentally obtained values.

## 4. Conclusion

In this study, a successful method for the preparation of Cibacron Blue F3GA-attached magnetic silica particles was developed. Due to the incorporation of the dye ligand into the magnetic silica particle matrix, these CB–F3GA attached magnetic silica particles exhibited high selectivity, fast adsorption kinetics, large binding capacity and outstanding reproducibility. It also suggested that in the selective isolation and enhancement of HSA from synthetic plasma protein, the given method could be expected another solution for the affinity of high amounts of protein.

The synthesized particles were characterized using disperse X–ray, vibrating sample magnetometry, SEM, FTIR, elemental analysis, and surface area measurement. The homogeneous composite average size of magnetic silica particles was ~7.8 μm. Adsorption exhibited a separation capacity of 34.9 mg/g for HSA at pH 5.5 buffer. Furthermore, the binding kinetic and adsorption isotherm models for the HSA adsorption parameter were described. Consequently, as consequence is seen that on a uniform polymer surface adsorption process is monolayer rate-limiting kinetics. The adsorption study was investigated by performing HSA adsorption from artificial plasma. After adsorption, albumin determination in plasma samples was carried out by the colorimetric method. With the data obtained, the amount of albumin adsorbed was determined as 48.6 mg/g. Considering the total amount of protein, protein removal from the artificial plasma was calculated to be approximately 97%. Thus, according to the results obtained, Cibacron Blue F3GA attached magnetic silica particles have become an adsorbent matrix to be taken as a reference for other studies by allowing simple, fast, inexpensive, and active purification of HSA with high capacity in HSA purification, as well as higher adsorption under the developed magnetic system.

## Supplementary Material

The following files are available as supplemental material on FTIR data, absorbance data, adsorption models, and kinetic model curves.

Figure S1 FTIR spectra of dye-attached magnetic silica particles (green) and magnetic silica particles (red).

Figure S2 Effect of the amount of Cibacron Blue F3GA on HSA adsorption.

Figure S3 Comparison of adsorption value studied in the presence of magnetic field: pH: 5.5, HSA concentration: 1.0 mg/mL, Particle amount: 25 mg, Temperature: 25 °C, Time: 2 h, Magnetic field: 25 mT.

Figure S4 Kinetic models; (a) the pseudo – first order and (b) the pseudo – second order.

Figure S5 (a) Langmuir model; (b) Freundlich model; (c) Comparison of experimental adsorption capacity.

## Figures and Tables

**Table 1:** The morphological and physical properties of the structures.

CV (%)	Average pore size (nm)	Pore volume (cc/g)	SSA (m^2^/g)
4.50	41.00	0.43	222.00

**Table 2:** Elemental analysis results.

Cibacron Blue F3GA concentration (mg/mL)	Amount of attached Cibacron Blue F3GA (mmol/g)
0.5	0.13
1.0	0.28
3.0	0.62
5.0	0.76

**Table 3:** The characteristic bands of silica particles.

	Wave number (cm^−1^)	Characteristic bands

Silica coated iron oxide	3293	Si – O – Si
	1645	O – H
	1053	Si – O – Si
	790–410	Fe – O

Cibacron Blue F3GA attached silica coated iron oxide	1594	C = N
	1355	S = O
	1057	C – Cl
	1033	S = O
	592–406	C – Cl

**Table 4:** Comparison of different types of adsorbents and albumin purification methods.

Method	Polymer material	Adsorption capacity	Sample	Ref.
Solid**–**phase extraction	PIL – modified graphene PIL – G/SiO_2_ nanocomposites	144.g mg/g	Bovin serum albumin	[32]
Proteomic analyses	Synthetic nanopockets on magnetic nanoparticle	21 mg/g	Human serum sample	[33]
Affinity chromatography	magnetic poly(vinyl alcohol) (mPVAL) beads	88.7 mg/g	Human serum albumin	[34]
Surface imprinting technique	Cobalt – molybdenum double-hydroxides surface-imprinted composite particles (Co – Mo LDHs @ MIPs)	423.37 mg/g	Bovine serum albumin	[35]
Dye**–**ligand affinity chromatography	CB – F3GA – attached magnetic silica particles	48.6 mg/g	Artificial Plasma	**This work**

**Table 5:** The pseudo – first – order and the pseudo-second-order kinetic models results.

Initial concentration	Experimental	Pseudo first order			Pseudo second order		
mg/mL	Q_eq_ (mg/g)	Q_eq_ (mg/g)	k_1_ (dk^−1^)	R^2^	Q_eq_ (mg/g)	k_2_ (g/mg. dk)	R^2^
1.50	31.22	4.71	0.0094	0.9589	38.16	0.0077	0.9802

**Table 6:** Langmuir and Freundlich adsorption model constants.

Experimental	Langmuir			Freundlich		
Q (mg/g)	Q_max_ (mg/g)	b (mL/mg)	R^2^	K_f_	n	R^2^
31.22	37.59	4.93	0.9782	18.17	1.17	0.9091
