# Supplementary figures and images for: Cibacron Blue F3GA ligand dye-based magnetic silica particles for the albumin purification
Source: Turk J Chem. 2023 Oct 10;47(5):1125–37. doi: 10.55730/1300-0527.3599 (PMC10760827; doi:10.55730/1300-0527.3599)

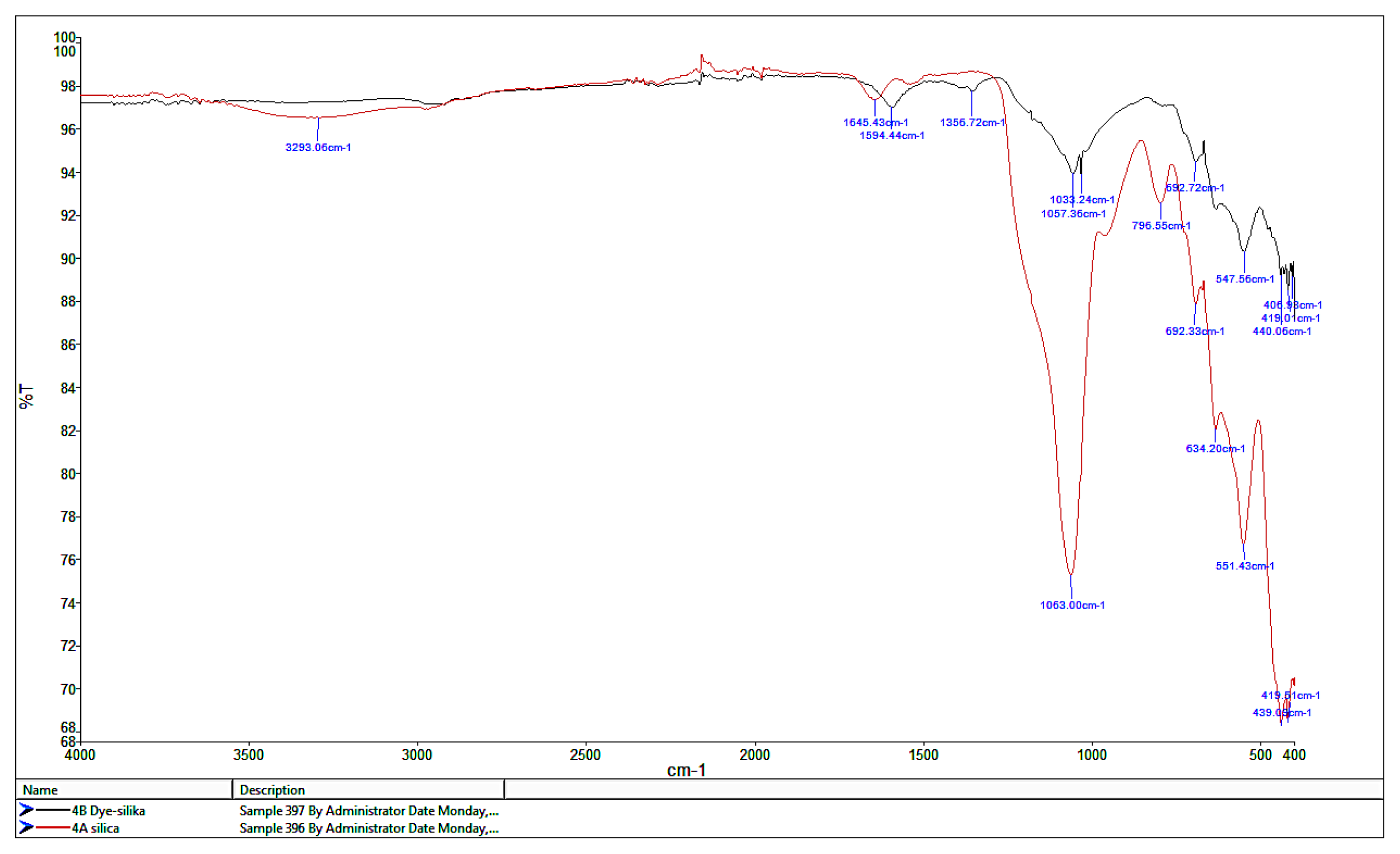

Supplement: Figure S1 — FTIR spectra of dye-attached magnetic silica particles (green) and magnetic silica particles (red). [file turkjchem-47-5-1125s1.tif]

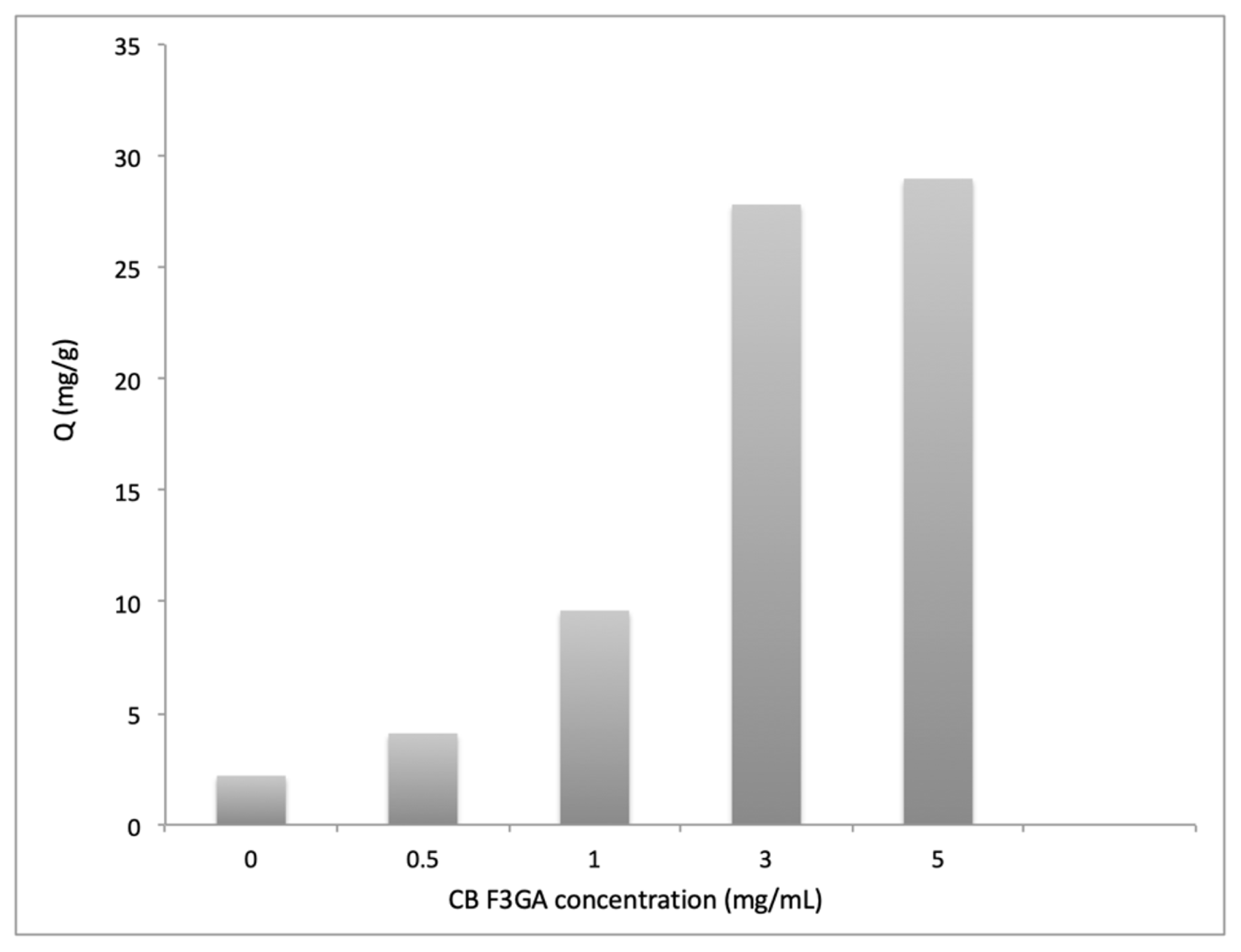

Supplement: Figure S2 — Effect of the amount of Cibacron Blue F3GA on HSA adsorption. [file turkjchem-47-5-1125s2.tif]

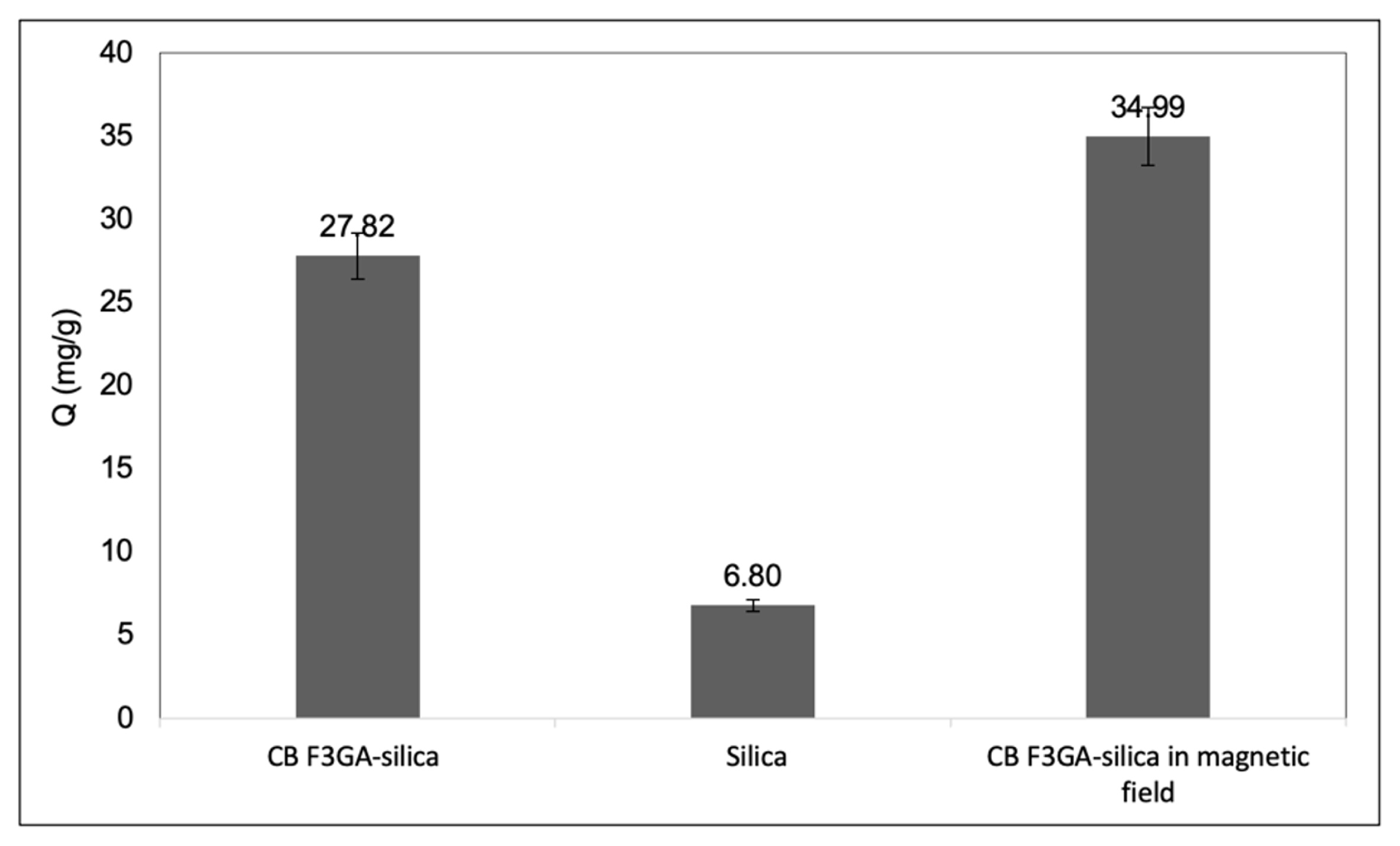

Supplement: Figure S3 — Comparison of adsorption value studied in the presence of magnetic field: pH: 5.5, HSA concentration: 1.0 mg/mL, Particle amount: 25 mg, Temperature: 25 °C, Time: 2 h, Magnetic field: 25 mT. [file turkjchem-47-5-1125s3.tif]

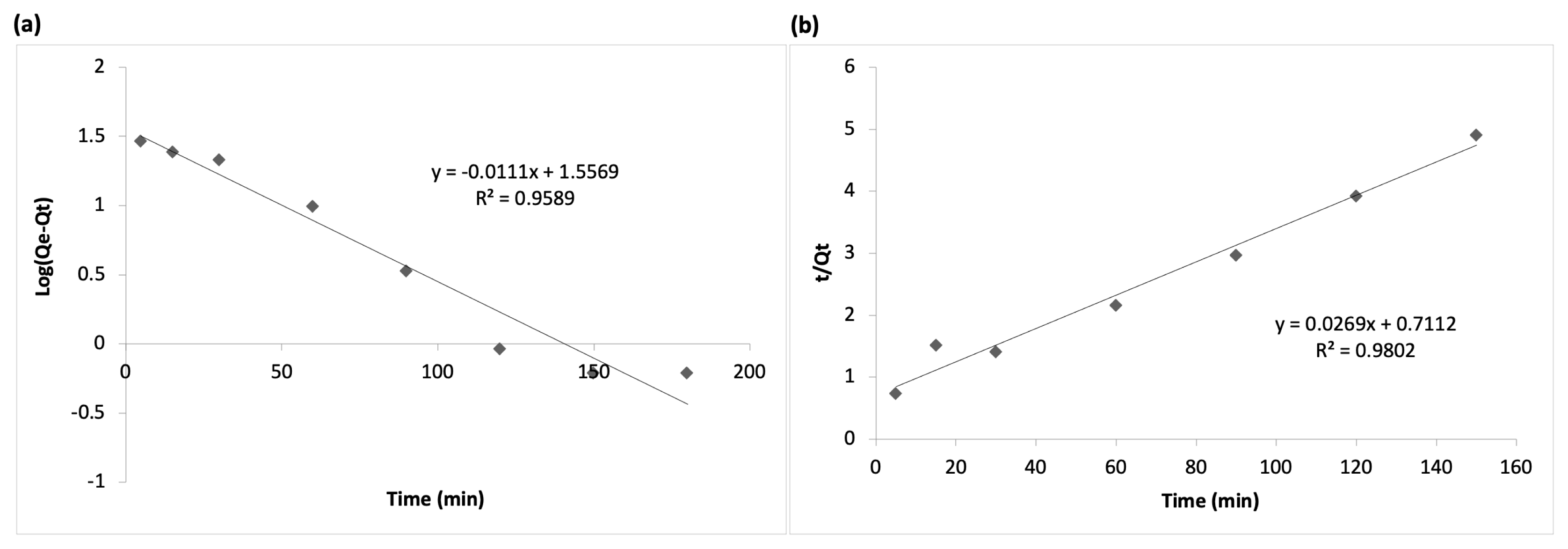

Supplement: Figure S4 — Kinetic models; (a) the pseudo – first order and (b) the pseudo – second order. [file turkjchem-47-5-1125s4.tif]

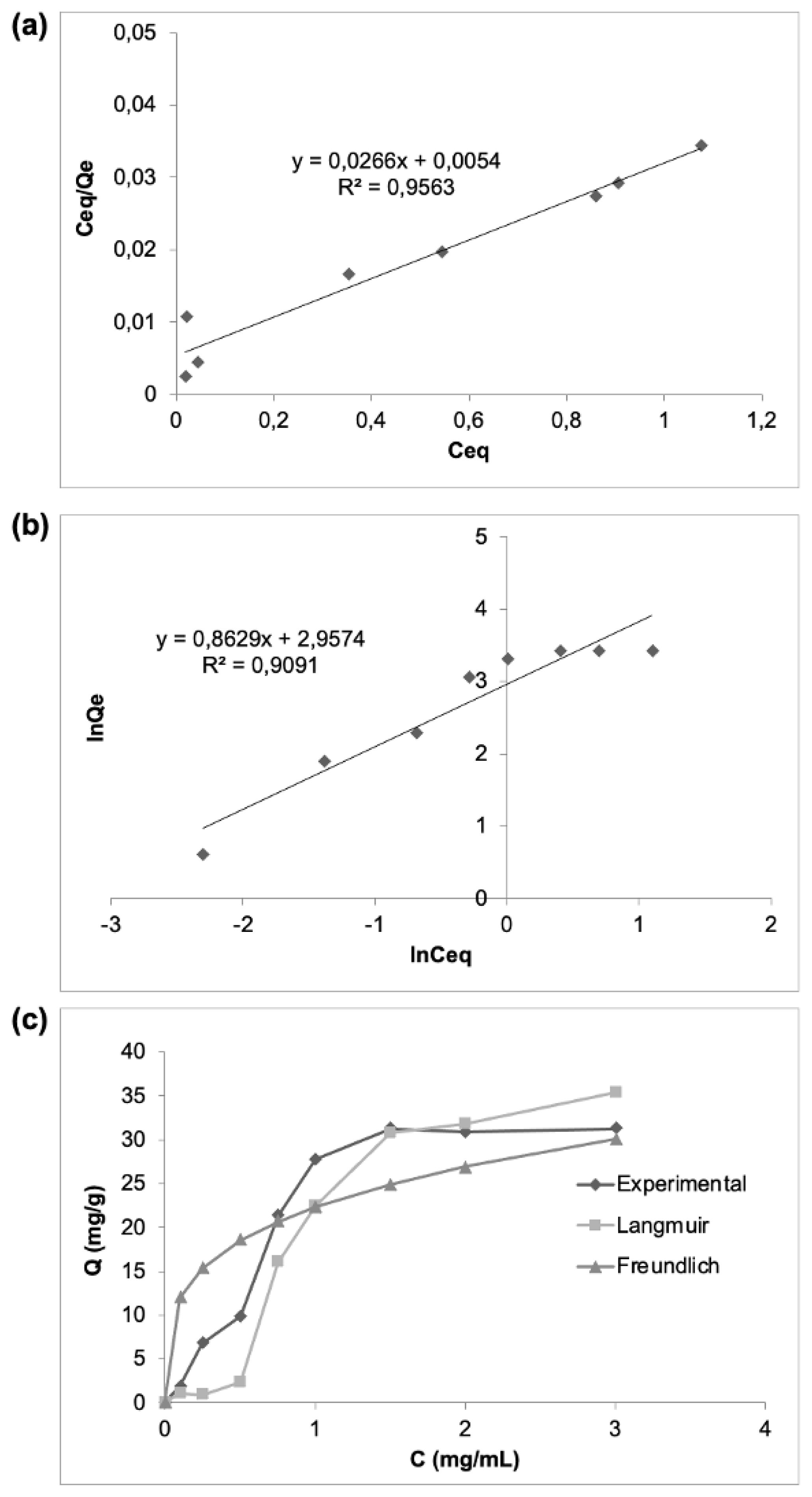

Supplement: Figure S5 — (a) Langmuir model; (b) Freundlich model; (c) Comparison of experimental adsorption capacity. [file turkjchem-47-5-1125s5.tif]
